# Neuronal carbonic anhydrase VII provides GABAergic excitatory drive to exacerbate febrile seizures

**DOI:** 10.1038/emboj.2013.160

**Published:** 2013-07-23

**Authors:** Eva Ruusuvuori, Antje K Huebner, Ilya Kirilkin, Alexey Y Yukin, Peter Blaesse, Mohamed Helmy, Hyo Jung Kang, Malek El Muayed, J Christopher Hennings, Juha Voipio, Nenad Šestan, Christian A Hübner, Kai Kaila

**Affiliations:** 1 Department of Biosciences, University of Helsinki, Helsinki, Finland; 2 Institute of Human Genetics, University Hospital Jena, Friedrich-Schiller-University Jena, Kollegiengasse 10, Jena, Germany; 3 Department of Neurobiology and Kavli Institute for Neuroscience, Yale University School of Medicine, New Haven, CT, USA; 4 Neuroscience Center, University of Helsinki, Helsinki, Finland

**Keywords:** carbonic anhydrase expression, chloride accumulation, GABA_A_ receptor, human brain, hyperthermia

## Abstract

Brain carbonic anhydrases (CAs) are known to modulate neuronal signalling. Using a novel CA VII (*Car7*) knockout (KO) mouse as well as a CA II (*Car2*) KO and a CA II/VII double KO, we show that mature hippocampal pyramidal neurons are endowed with two cytosolic isoforms. CA VII is predominantly expressed by neurons starting around postnatal day 10 (P10). The ubiquitous isoform II is expressed in neurons at P20. Both isoforms enhance bicarbonate-driven GABAergic excitation during intense GABA_A_-receptor activation. P13–14 CA VII KO mice show behavioural manifestations atypical of experimental febrile seizures (eFS) and a complete absence of electrographic seizures. A low dose of diazepam promotes eFS in P13–P14 rat pups, whereas seizures are blocked at higher concentrations that suppress breathing. Thus, the respiratory alkalosis-dependent eFS are exacerbated by GABAergic excitation. We found that CA VII mRNA is expressed in the human cerebral cortex before the age when febrile seizures (FS) occur in children. Our data indicate that CA VII is a key molecule in age-dependent neuronal pH regulation with consequent effects on generation of FS.

## Introduction

GABA_A_-receptor (GABA_A_R)-mediated signalling has a wide spectrum of functions in neurons and neuronal networks (Farrant and Kaila, 2007). Paradoxically, intense activation of GABAergic synapses in mature neurons may directly promote rather than suppress neuronal excitation (Alger and Nicoll, 1982; Kaila et al, 1997). We and others have previously demonstrated that, in the rat hippocampus, GABA_A_R-mediated excitation is strictly dependent on the continuous replenishment of neuronal HCO_3_^−^ by cytosolic carbonic anhydrase (CA) activity and suppressed by membrane-permeant inhibitors of CA (Staley et al, 1995; Kaila et al, 1997; Fujiwara-Tsukamoto et al, 2007; Viitanen et al, 2010). Whether HCO_3_^−^-dependent GABAergic excitation has a role in the generation of seizures *in vivo* is not known.

CAs affect the kinetics and amplitudes of pH transients in distinct intra- and extracellular compartments (Chesler, 2003; Casey et al, 2010; Ruusuvuori and Kaila, 2013) and can thereby influence the function of a wide variety of proton-sensitive membrane proteins involved in neuronal signalling such as GABA_A_Rs (Pasternack et al, 1996; Wilkins et al, 2005), *N*-methyl-D-aspartate receptors (Traynelis et al, 2010; Makani et al, 2012), acid-sensing ion channels (Waldmann et al, 1997), cation channels (Munsch and Pape, 1999; Williams et al, 2007; Enyedi and Czirjak, 2010) and gap junctions (Spray et al, 1981). In addition, pH transients can have modulatory actions on ion channels mediated by changes in fixed charges on the plasmalemmal surface (Velisek et al, 1994; Hille, 2001, see pp 653–654).

CA inhibitors such as acetazolamide and its more lipophilic derivatives have a long history as anticonvulsants, but the molecular targets and mechanisms of action at the neuronal network level are still poorly understood (Thiry et al, 2007; Supuran, 2008). The available inhibitors are not selective with respect to the 13 catalytically active isoforms that have been identified so far (Supuran, 2008) and may thus exert their actions within and outside the CNS. It is possible that their effects on neuronal functions are at least partly mediated by nitric oxide (Aamand et al, 2011) or calcium-activated potassium channels (Pickkers et al, 2001).

Using a novel CA VII knockout (KO) mouse as well as a CA II KO (Lewis et al, 1988) and a novel CA II/VII double KO, we show here that CA II and CA VII are the only cytosolic isoforms present in both somata and dendrites of mature hippocampal CA1 pyramidal neurons, with expression of CA VII commencing at postnatal day 10 (P10) and that of CA II at around P20. Immunoblots from glial or neuron/glial cultures showed that CA VII expression is restricted to neurons while CA II is present in both types of cells. After P30, the two isoforms are similarly effective in promoting GABAergic excitation.

In line with our previous data on rats (Schuchmann et al, 2006), hyperthermia generated a similar respiratory alkalosis in both wild-type (WT) and CA VII KO mice at P13–14. Typical experimental febrile seizures (eFS) with cortical electrographic seizure activity were observed in WT mice but not in the CA VII KOs. Diazepam potentiated excitatory GABAergic transmission *in vitro*, and a low concentration of the drug reduced the time to eFS onset in P14 rats *in vivo*. Consistent with a role in human febrile seizures (FS), we found that CA VII is present in the human cortex and hippocampus already at the perinatal stage, well before 6 months of age when FS are typically first observed (Berg and Shinnar, 1996; Stafstrom, 2002). Thus, CA VII is a key molecule in age-dependent neuronal pH regulation with consequent effects on generation of eFS.

## Results

### Generation of the CA VII KO mouse

**Figure 1:**
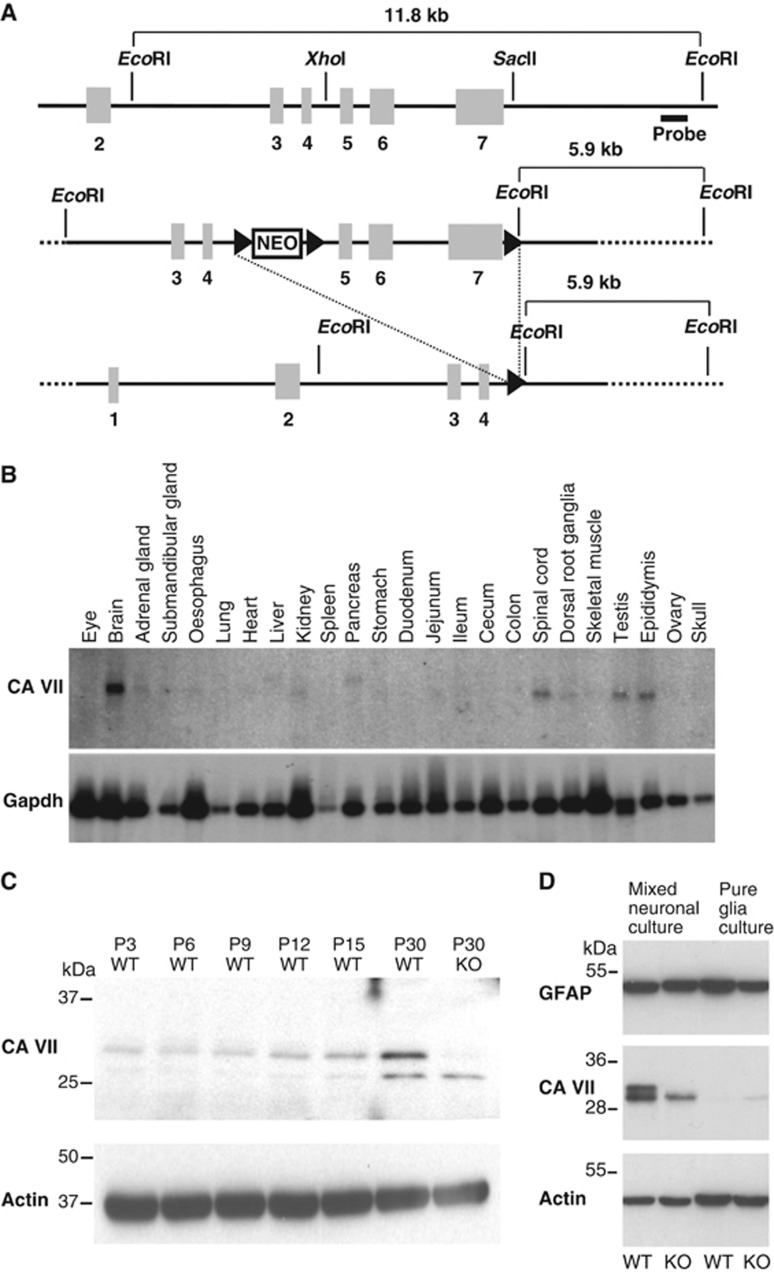
Generation of CA VII-deficient mice. (**A**) Targeting strategy for the *Car7* locus. Endogenous *Car7* locus (top). Targeting construct (middle). Targeted locus after Cre-mediated recombination (bottom). Exons are indicated as grey boxes and loxP sites as arrowheads. (**B**) Multiple tissue northern blot for CA VII. In the P56 mouse, CA VII is prominently expressed in the central nervous system including brain and spinal cord. *Gapdh* served as the loading control. (**C**) Developmental profile of CA VII protein expression. Western blot analysis of protein lysates of whole hippocampi shows increasing CA VII levels from P3 to P30. The band of the appropriate size (∼30 kDa) was absent in protein lysates from KO tissue. (**D**) In western blots from protein lysates of cultured cells, CA VII was detected in mixed glia/neuron cultures but not in glia cell cultures. The presence of astrocytes in all cultures was confirmed by detection of GFAP. Actin served as the loading control in **C** and **D**.

We floxed exons 5–7 of the *Car7* gene (Figure 1A), and homologous recombination was verified by Southern blot analysis with a 3′ probe using an additional *EcoR*I site in the targeted allele. In two independent correctly targeted ES cell clones, we transiently expressed Cre-recombinase. The resulting subclones were screened for deletion of the DNA fragment flanked by the outer loxP sites. Two independent subclones were then injected into blastocysts and transferred to foster mice. The resulting chimeras were mated with C57BL/6 mice and produced offspring to heterozygous KO mice. Homozygous KO mice were born from heterozygous matings in the expected Mendelian ratio (∼25%) and did not display any obvious phenotype within the age range used in the present experiments (up to P56, where P0 refers to the day of birth) or in the breeding animals used for about 1 year.

### Expression patterns of CA VII mRNA and protein

Northern blot analysis in adult mouse tissues showed that CA VII is prominently expressed in the mature brain (Figure 1B). To detect CA VII at the protein level, a novel polyclonal antiserum against an epitope of murine CA VII was raised in rabbits and affinity-purified. In protein lysates of adult WT whole hippocampi, this antibody detected a band of an appropriate size of ∼30 kDa, which was absent from protein lysates prepared from the hippocampi of CA VII KO mice (Figure 1C). In WT mice, CA VII protein abundance increased in the hippocampus during postnatal maturation. Immunoblots on CA VII from lysates of glial and mixed neuron/glia cultures showed that, unlike CA II, CA VII is expressed in neurons but not in glia (Figure 1D). Using an antibody that is not validated in CA VII KO tissue, Bootorabi et al (2010) reported expression of CA VII in numerous mouse tissues including skeletal muscle and liver, while no signal was seen in the CNS. These data are in stark contrast with the mRNA expression analysis shown in Figure 1B and also with immunoblots based on our antibody in muscle and liver where no CA VII protein was detected (Supplementary Figure S1).

### CA VII is the first functional cytosolic isoform expressed in somata of CA1 pyramidal neurons

**Figure 2:**
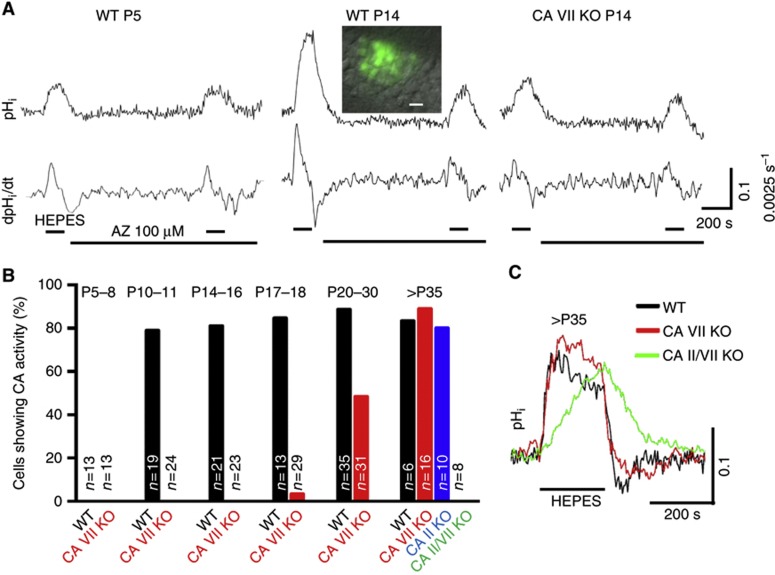
Development of cytosolic CA activity in mouse CA1 pyramidal neurons is based on sequential expression of isoforms VII and II. (**A**) Original single-cell pH_i_ traces and their time derivatives from P5 and P14 WT and P14 CA VII KO neurons (baseline pH_i_ 7.21, 7.13 and 7.11, respectively). Superfusion with CO_2_/HCO_3_^−^-free HEPES solution (upper horizontal bars) evoked an intracellular alkalinization, which in P14 WT was large and suppressed by 100 μM acetazolamide (AZ, lower horizontal bar), indicating the presence of CA activity (see Supplementary Materials and methods). The possible effect of AZ on extracellular CAs was excluded by adding 10 μM benzolamide (a poorly permeant CA inhibitor). Inset shows an overlay of the BCECF fluorescence signal and Dodt gradient contrast image of CA1 pyramidal neurons in P14 WT (scale bar 10 μm). (**B**) Summary of the results obtained using the cytosolic CA activity detection method shown in A and quantified as the percentage of cells showing cytosolic CA activity. Data from CA II KO and CA II/VII KO neurons were obtained only at >P35. The number of cells tested is indicated for each bar. The animal numbers for WT mice at the different age points was *n*=3 (P5–8), *n*=2 (P10–11), *n*=3 (P14–16), *n*=2 (P17–18), *n*=2 (P20–30) and *n*=2 (>P35); for CA VII KO mice the numbers were *n*=3 (P5–8), *n*=2 (P10–11), *n*=5 (P14–16), *n*=3 (P17–18), *n*=4 (P20–30) and *n*=2 (>P35); and for CA II KO and CA II/CA VII KO mice the number was *n*=2 (>P35). (**C**) Typical single pyramidal neuron pH_i_ responses evoked by withdrawal of CO_2_/HCO_3_^−^ (horizontal bar, HEPES) in slices from >P35 WT, CA VII KO and CA II/VII KO mice. The rate of rise and the amplitude of the alkaline shift are identical in WT (P39, baseline pH_i_ 7.01) and CA VII KO neurons (P40, 7.06), whereas alkalinization develops much more slowly in the CA II/VII KO (P46, 7.04).

Our previous results on rats showed a temporal coincidence between the developmental upregulation of CA VII mRNA and enhanced CA activity probed by CA inhibitors in pyramidal neurons (Ruusuvuori et al, 2004). Because the available cytosolic CA inhibitors are not isoform specific, these data do not exclude the possible presence of other neuronal CA isoforms. To overcome this problem, we examined the emergence of CA activity in somata of the hippocampal CA1 pyramidal neurons using BCECF fluorescence recording of intracellular pH (pH_i_) in slices from neonatal, juvenile and adult WT and the three CA transgenic mice (CA VII KO, CA II KO and CA II/VII double KO) (Figure 2).

An abrupt developmental onset of cytosolic CA activity was detected in WT neurons at P10. The alkaline shifts measured in the somatic area upon CO_2_/HCO_3_^−^ withdrawal became faster and larger and the membrane-permeant CA inhibitor acetazolamide caused a clear suppression of the maximum rate of the alkalinization in 81% of P10–18 neurons (*n*=53) (Figure 2A and B). In sharp contrast to WT, functional cytosolic CA was not detected in CA VII KO neurons at this age. The alkalinization was slow and insensitive to acetazolamide in all of the <P17 CA VII KO neurons (*n*=60) and in 28 of 29 P17–18 CA VII KO neurons, similar to the pH_i_ response in WT neurons before the onset of CA expression (*n*=13, WT neurons <P10) (Figure 2A and B). These results provide firm evidence for the conclusions that (i) the developmental upregulation of CA activity in the somatic area of mouse hippocampal CA1 pyramidal neurons at P10 is solely attributable to CA VII, and thus (ii) this is the *only cytosolic isoform* that shows catalytic activity in the P10–18 time window.

The expression of CA VII caused a small change in the baseline pH_i_ (Supplementary Figure S2), which can be readily explained by production of CO_2_ within the slice (Voipio and Kaila, 1993).

### Upregulation of cytosolic CA activity in somata of >P18 CA VII KO neurons is attributable to CA II

Surprisingly, an upregulation of CA activity was observed in neurons from CA VII KO mice at around P20 (Figure 2B and C). CA activity was observed in about 50% of >P18 neurons, and after P35 the percentage of CA VII KO cells showing catalytic activity was similar to that in WT.

Measurements of pH_i_ in CA II/VII double KO neurons demonstrated that they were completely devoid of cytosolic CA activity even after P35. In contrast to this, CA activity was detected in a roughly similar fraction in WT, CA VII KO and CA II KO neurons after P35 (83, 88 and 80%). After P25, the baseline of somatic pH_i_ was more acidotic compared with P14–15 neurons from WT and CA VII KO, and there was no longer a difference in the mean resting pH_i_ between the two genotypes (Supplementary Figure S2), implying that the presence of both CA VII and CA II, or of CA II alone, had an identical effect on baseline pH. Thus, experiments with the three KO transgenic mice show that the cytosol of mature CA1 pyramidal neuron somata in WT mice is endowed with two CA isoforms, CA II and CA VII.

### Depolarizing dendritic GABA responses are promoted by CA II and CA VII

**Figure 3:**
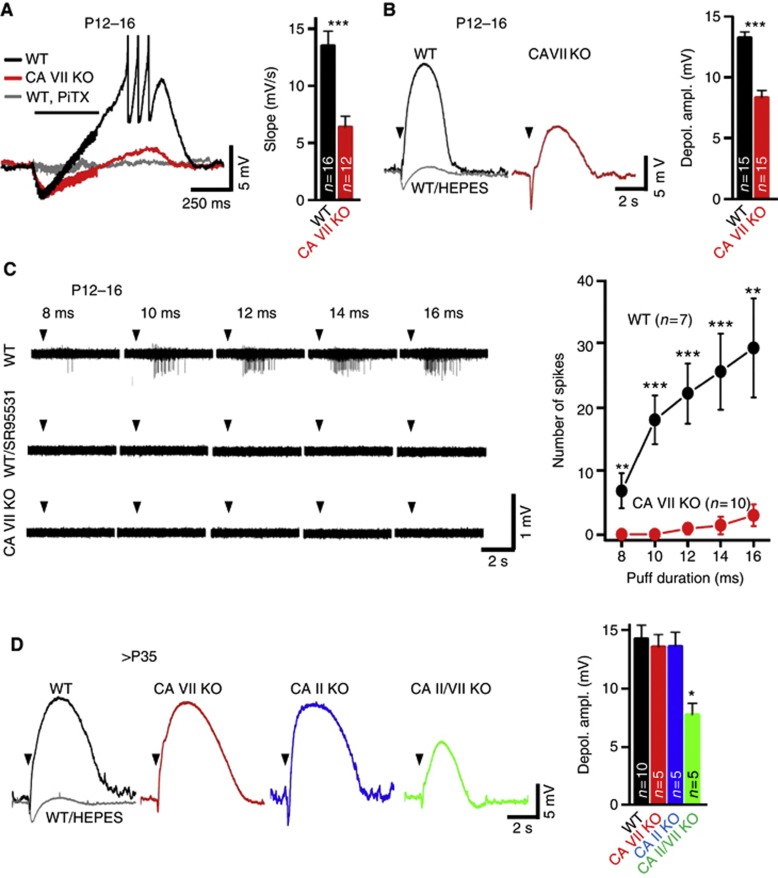
CA VII and CA II similarly enhance GABA_A_R-mediated depolarization in CA1 pyramidal neurons. (**A**) Electrical stimulation (40 pulses, 100 Hz at *stratum radiatum/stratum lacunosum-moleculare* (*sr/slm)* border (horizontal bar)) in the presence of AP5/CNQX/CGP55845) evokes an excitatory GABAergic response in WT, but not in CA VII KO neurons. Picrotoxin blocked the biphasic response (PiTX 90 μM; *n*=3/genotype). The mean initial slope of the depolarizing shift was faster in WT than in CA VII KO (13.53±1.27 versus 6.39±0.96 mV/s, *P*<0.001, Student’s *t*-test). The resting membrane potential of WT and CA VII KO neurons did not differ (−71.0±1.1 versus −72.3±1.4 mV, respectively; *n*=15 neurons for both genotypes, *P*=0.47, Student’s *t*-test). (**B**) Microinjection of GABA (5 mM for 100 ms, *sr/slm* border in the presence of CGP55845/TTX, arrowheads) induced a pronounced depolarization in WT neurons (black) that was abolished in the absence of CO_2_/HCO_3_^−^ (grey). The depolarization was smaller in CA VII KO (red). Bar diagram illustrates GABAergic mean peak depolarization in P12–16 WT and CA VII KO (13.3±0.5 versus 8.3±0.6 mV, *P*<0.001, Student’s *t*-test). (**C**) In the presence of AP5/CNQX/CGP55845, microinjection of GABA (5 mM, *sr/slm* border) triggered SR 95531-sensitive field potential spikes in WT slices already upon 8-ms injection, while even 16-ms injection evoked hardly any spikes in CA VII KO (left). Mean number of spikes was plotted against GABA puff duration in P12–16 WT and CA VII KO (*P*<0.01 and *P*<0.001, Student’s *t*-test; right). (**D**) After P35, depolarizations upon GABA microinjection (arrowheads) in the presence of CGP55845/TTX were indistinguishable in WT (black), CA VII KO (red) and CA II KO (blue) but smaller in CA II/VII KO (green). The depolarization in WT was abolished in the absence of CO_2_/HCO_3_^−^ (grey, *n*=5). Bar diagram summarizes mean peak depolarization for each genotype (right; ANOVA, Bonferroni). *P<0.05, **P<0.01 and ***P<0.001. Error bars denote s.e.m. and *n* is the number of slices (**C**) or cells tested (one cell per slice) (**A**, **B** and **D**).

Next, we studied the developmental expression patterns of CA isoforms in the dendrites of pyramidal cells. To this end, we first used whole-cell recordings from CA1 pyramidal neurons in P12–16 WT and CA VII KO slices and examined GABAergic synaptic responses. Biphasic GABAergic responses were evoked with high-frequency stimulation applied at the border of *stratum radiatum (sr) and stratum lacunosum-moleculare (slm)* in the presence of ionotropic glutamate receptor and GABA_B_R antagonists. In both WT and CA VII KO neurons, this stimulation evoked a biphasic response consisting of an initial hyperpolarization followed by a prolonged depolarization, both of which were abolished by picrotoxin (Figure 3A). The gradual positive shift has been shown to closely reflect the HCO_3_^−^-driven conductive net uptake of Cl^−^ (Viitanen et al, 2010). Importantly, the slope of the depolarizing shift measured under current clamp conditions was considerably slower in CA VII KO than in WT neurons (Figure 3A). This indicates that the dendritic HCO_3_^−^ -dependent net uptake of Cl^−^ is strongly facilitated by CA activity in P12–P16 WT neurons. Measurements under voltage clamp conditions showed that the apparent charge transfer associated with single inhibitory post-synaptic currents was not different between the two genotypes (Supplementary Figure S3A).

In agreement with previous work on rat slices (Kaila et al, 1997; Ruusuvuori et al, 2004), the long-lasting GABAergic depolarization was able to induce action potential firing in WT neurons (Figure 3A). In striking contrast to this, action potentials were not observed in any of the 12 P12–16 CA VII KO neurons tested. A confounding factor in these experiments is a possible difference in the intrinsic excitability of WT versus CA VII KO neurons. We examined this by measuring the input resistance and the minimum current injection needed to trigger spiking (rheobase) in whole-cell mode, but no difference was found between neurons from WT and CA VII KO mice (Supplementary Figure S3B).

The role of the two cytosolic CA isoforms in the generation of dendritic depolarizing GABA_A_ responses was examined using whole-cell recordings from CA1 pyramidal neurons in WT and CA VII KO slices at P12–16 (Figure 3B). Pressure microinjection of GABA at the border of *sr/slm* gave rise to biphasic membrane potential responses, where the depolarizing phase was much smaller in CA VII KOs than in WT. As expected, the depolarizing phase was selectively blocked in the absence of CO_2_/HCO_3_^−^ (in the HEPES buffered-solution).

That GABA is excitatory and induces neuronal firing in a CA VII-dependent manner in intact P12–16 WT neurons was clearly seen in field potential recordings (Figure 3C). In all slices from P12–16 WT animals, pressure injection of GABA at the border of *sr/slm* induced spiking and the number of spikes increased with the duration of the GABA puff (8 ms: 6.9±2, 10 ms: 18.0±3.8, 12 ms: 22.1±4.7, 14 ms: 25.6±6.0 and 16 ms: 29.3±7.8). Spiking was fully blocked by the GABA_A_-receptor antagonist SR 95531 (gabazine). In contrast, in slices from CA VII KO mice, GABA application evoked hardly any spiking even with the longest puff.

When examining intracellular GABA responses after P35 using GABA pressure injection as described above, no difference was observed between the WT, CA II KO and CA VII KO neurons, indicating that both CA VII and CA II activity can promote depolarizing GABA responses in dendrites (Figure 3D, see also Kaila et al, 1997). In agreement with this, the dendritic GABA-evoked depolarization was much smaller in neurons from >P35 CA II/VII double KO animals (Figure 3D), and similar to that seen in P12–P16 CA VII KO neurons (Figure 3B). The presence of the attenuated but not fully abolished response in the double KO slices is consistent with the fact that, while replenishment of HCO_3_^−^ by CA is rate-limiting for the GABAergic depolarization, the steady-state HCO_3_^−^ concentration of about 10 mM (pH_i_=6.9, pH_o_=7.3) is sufficient for a minor depolarizing action.

### Lack of electrographic FS in CA VII KO mice

The seizure susceptibility of a large number of transgenic mice, including knock-in animals with gene variants that promote FS and related epileptiform syndromes in humans, has been tested using eFS triggered by exposure to hyperthermia (Meisler et al, 2001; Dube et al, 2005; van Gassen et al, 2008; Wimmer et al, 2010; Hill et al, 2011). The distinct developmental expression profiles of CA VII and II made it possible to specifically examine the role of isoform VII in the generation of eFS using P13–14 WT and CA VII KO mice. This developmental stage is considered relevant for comparisons with humans, in whom the peak incidence of FS is reached at the age of 18 months (Berg and Shinnar, 1996; Stafstrom, 2002). eFS were evoked using a pre-heated chamber as described before (Schuchmann et al, 2006).

As expected on the basis of previous work (Meisler et al, 2001; Dube et al, 2005; van Gassen et al, 2008), WT mice (*n*=4) when exposed to hyperthermia first showed normal explorative behaviour (score 0 in a behavioural scale of mouse eFS (van Gassen et al, 2008)) followed by hyperthermia-induced hyperactivity and escape responses (score 1). Thereafter, immobility and ataxia (score 2) were seen, after which seizures progressed to more severe manifestations including shaking, clonic seizures of one or more limbs (score 3), culminating in continuous tonic-clonic seizures (score 4) at a rectal temperature of 41.9±1.1°C. Remarkably, none of the CA VII KO mice (*n*=8) showed progression of seizures beyond score 3 at similar temperature levels of 42.5±0.5°C. Moreover, the seizure activity in the KO was atypical of eFS also in that it was interspersed with periods during which coordinated behaviour patterns such as walking and exploratory activity were seen, which never occurred in WT mice once seizures had progressed to the clonic stage.

**Figure 4:**
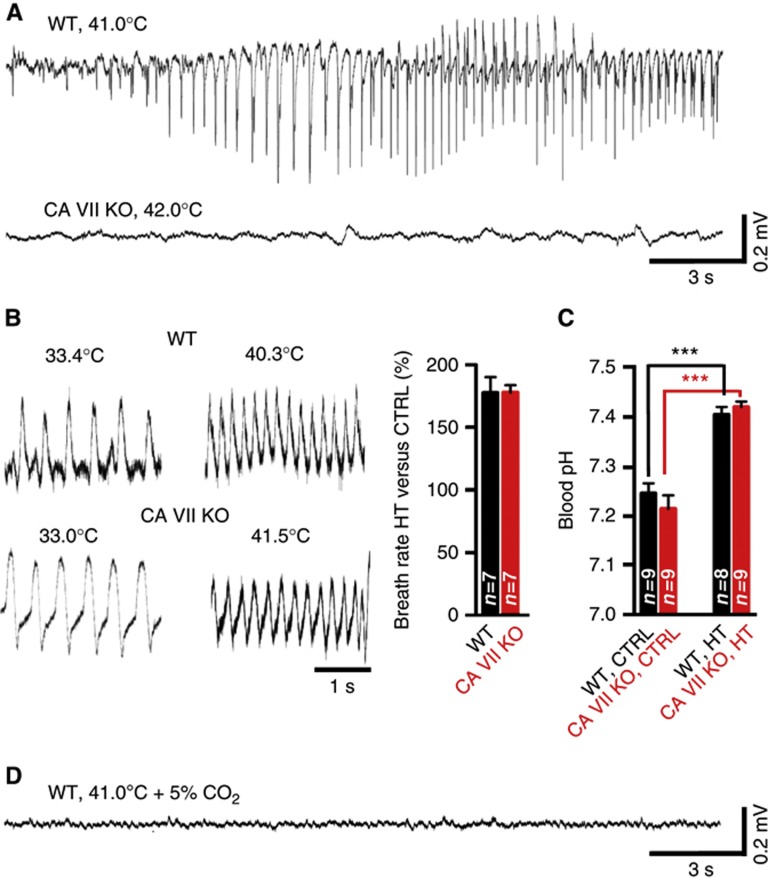
CA VII promotes experimental febrile seizures in P13–14 mice. (**A**) Representative raw traces of epidural EEG recordings during hyperthermia (HT) from a WT and a CA VII KO mouse. Seizures were reliably induced in WT (*n*=9) but not in CA VII KO (*n*=7). (**B**) Specimen recordings of breath rate from a WT and a CA VII KO mouse. The baseline breath rate (WT 153±11 breaths/min and CA VII KO 157±8 breaths/min) was increased during HT in both genotypes (WT 272±28 breaths/min and CA VII KO 279±12 breaths/min). The bar diagram summarizes the mean increase in breath rate in WT (177±12%) and CA VII KO mice (178±6%, *P*=0.98). (**C**) Hyperthermia caused a comparable alkalosis in blood pH in both WT and CA VII KO mice. The blood pH did not differ between the two genotypes under control conditions (*P*=0.37) or during HT (*P*=0.44). (**D**) Electrographic seizures were not detected in WT mice when hyperthermia was induced in the continuous presence of 5% CO_2_ (*n*=5). Error bars denote s.e.m., all *P*-values are based on Student’s *t*-test (***P<0.001), and animal number is given in bar diagrams. Rectal temperatures are given above the traces.

Given the above qualitative difference in the behavioural hyperthermia effects between the WT and CA VII KO, we used EEG to gain more information on the underlying mechanisms. Epidural EEG recordings showed that, in nine out of nine WT mice, electrographic seizures commenced within 24±4 min after the onset of hyperthermia (Figure 4A). At this time point, the rectal temperature was 40.9±0.4°C. The seizures were easily recognized as large-amplitude bursts of spikes (150–600 μV) with a frequency of about 2–4 Hz and duration of 10–60 s, which, notably, occurred mainly during periods of behavioural arrest as also reported by others (Dube et al, 2000). Because we have previously shown that a respiratory alkalosis is a major trigger of eFS (Schuchmann et al, 2006, 2011), we also measured the breath rates as well as blood pH values in the two genotypes. At the time of eFS onset in the WT, the breath rate had increased to 177±12% (Figure 4B). In striking contrast to the WT, no electrographic seizures were observed in seven out of seven CA VII KO animals that were examined (Figure 4A). The rectal temperature (41.3±0.4°C) and breath rate at 24 min after the onset of hyperthermia in CA VII KO animals were similar to those in WT animals (Figure 4B). Importantly, direct measurements of blood pH showed that hyperthermia induced a respiratory alkalosis with identical magnitude in both genotypes (Figure 4C). These data indicate that a suppression of systemic respiratory alkalosis does not explain the absence of eFS in the CA VII KO mice. Consistent with our earlier findings (Schuchmann et al, 2006), the generation of both behavioural and cortical electrographic eFS in WT mice were fully prevented when hyperthermia was induced in the continuous presence of 5% CO_2_ in air (Figure 4D). Finally, measurements of blood electrolytes showed no genotype-specific differences in control conditions or under hyperthermia (Supplementary Figure S4).

**Figure 5:**
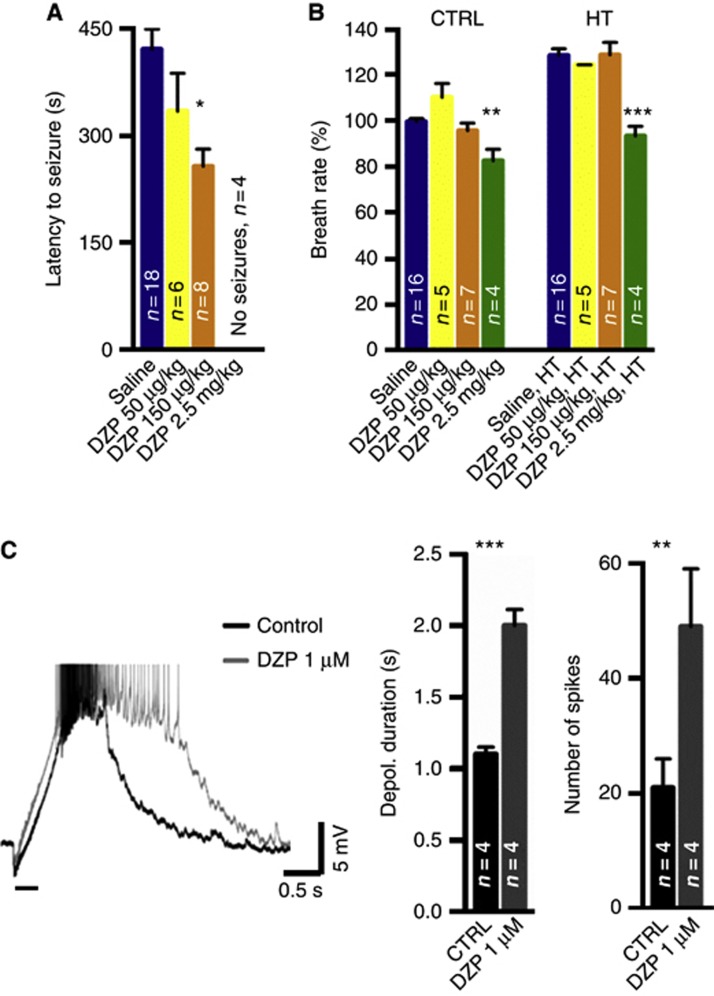
GABA_A-_receptor-mediated modulation of experimental febrile seizures. (**A**) Diazepam (DZP, 150 μg/kg) reduced the latency to seizure onset in P14 rat pups (*P*<0.05). There was no statistical difference between 50 μg/kg and saline-injected animals. DZP 2.5 mg/kg completely blocked the seizures. DZP was given intraperitoneally 15 min before hyperthermia (HT) onset. (**B**) Diazepam given at 150 μg/kg did not affect the baseline breath rate, and HT induced an analogous increase in the breath rate in saline- and diazepam-injected rats (*P*>0.05). At a higher concentration (2.5 mg/kg), DZP suppressed breathing significantly (*P*<0.01) and HT caused only a small increase in the breath rate. (**C**) In slice preparation, DZP (1 μM) potentiated the high-frequency stimulation (40 pulses at 100 Hz, horizontal bar) -evoked GABA_A_R-mediated depolarization. Sample trace from a slice from a P14 rat pup where stimulation to the border of *stratum radiatum* and *stratum lacunosum-moleculare* was given in the presence of AP5, CNQX and CGP 55845. The bar diagrams summarize the effects of DZP on the duration of the depolarization (time to half-maximum) and the number of action potentials associated with the depolarizing phase. *P*-values (*P<0.05, **P<0.01 and ***P<0.001) are based on ANOVA, Bonferroni (**A**, **B**) or Student’s *t*-test (**C**), and error bars denote s.e.m. The number of animals (**A**, **B**) or cells (**C**) is indicated in bar diagrams.

The above data raise the intriguing question whether CA VII-dependent GABAergic excitation contributes to the generation of cortical electrographic eFS. Hence, we performed behavioural experiments on P14 rat pups to examine whether enhancing GABA_A_R signalling by low doses of diazepam would facilitate the triggering of eFS. When diazepam was given at a very low dose (50 μg/kg intraperitoneally), latency to seizure onset decreased but the difference was not statistically significant from that seen in saline-injected animals (Figure 5A). However, a dose of 150 μg/kg resulted in a significant decrease in seizure latency. Importantly, neither of these two doses had a detectable effect on the baseline breath rate, and hyperthermia was associated with a similar increase in breath rate in rats injected with 50–150 μg/kg and in those injected with saline (Figure 5B). As could be expected (Hirtz and Nelson, 1983; Laorden et al, 1990), a higher dose of diazepam (2.5 mg/kg) prevented seizure generation in four of four rats tested, while the saline-injected control animals showed robust eFS (Figure 5A). At this concentration, diazepam suppressed the breath rate under control conditions and, under hyperthermia, only a modest increase in breathing was seen in the presence of the drug (Figure 5B).

In order to further examine the hypothesis that CA VII-dependent GABAergic excitation facilitates the generation of eFS, we evoked pharmacologically isolated excitatory GABAergic responses in P14 rat hippocampal slices. Consistent with the above hypothesis, the depolarizing phase as well as spiking evoked by the high-frequency stimulation was considerably enhanced by application of 1 μM diazepam (Figure 5C), with a maximum effect seen after 10 min of drug application.

As will be discussed below, the above *in vivo* and *in vitro* data as a whole suggest that eFS are facilitated by GABAergic excitation and that a low concentration of diazepam potentiates these effects by a selective action on GABA_A_Rs (Eghbali et al, 1997). The suppression of eFS by a high dose of diazepam is readily explained by the suppression of breathing (Figure 5B).

### Spatiotemporal expression patterns of human CA VII in the developing brain

**Figure 6:**
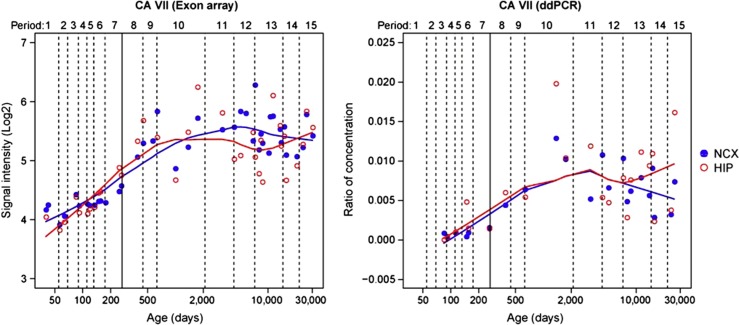
CA VII expression during human brain development. Line plots show the log_2_-transformed exon array signal intensity (left), and the normalized ratio of absolute copy number of CA VII to *Gapdh* by droplet digital PCR (ddPCR, right) in the neocortex (NCX) and hippocampus (HIP) from the early fetal period to late adulthood. The solid line between periods 7 and 8 separates the prenatal from the postnatal period.

We used exon array analysis and droplet digital PCR to examine the developmental expression of CA VII in the human neocortex and hippocampus (see Supplementary Materials and methods). Our previous study on the human brain transcriptome identified 29 modules corresponding to specific spatiotemporal expression patterns by weighted gene co-expression network analysis (Kang et al, 2011). Here, it is of much interest that, among them, CA VII was classified as a member of M2 (Supplementary Figure S5), a main module including 2745 genes, which is interpreted as a module related to synaptic transmission based on functional annotations using gene ontology (Kang et al, 2011). Our present data show that CA VII expression is markedly increased in the human neocortex and hippocampus during the perinatal period and sustained until late adulthood (Figure 6). Thus, an association between CA VII and epileptiform syndromes is not excluded at any stage of human neocortical and hippocampal development.

## Discussion

Distinct CA isoforms exert a profound modulatory influence on the functions of a wide variety of voltage-gated channels, excitatory and inhibitory synapses and gap junctions (see Introduction). Because isoform-specific CA inhibitors are not available, we used gene disruption of two cytosolic isoforms, CA II and CA VII, separately and in combination to demonstrate the following: (i) CA activity is fully attributable to these two isoforms in the somata and dendrites of mature mouse CA1 pyramidal neurons; (ii) the two isoforms are sequentially expressed in the developing hippocampus, with expression of CA VII commencing at P10 and that of CA II at around P20; (iii) in contrast to CA II, CA VII is not expressed in glia; (iv) both isoforms are able to promote HCO_3_^−^-dependent GABAergic depolarization and excitation triggered by intense GABA_A_R activation in mature neurons. This kind of information is crucial for understanding the mechanisms of pH-dependent modulation of neuronal and neuronal network functions in the developing and mature brain (Kaila and Ransom, 1998; Schuchmann et al, 2009; Casey et al, 2010; Tolner et al, 2011; Magnotta et al, 2012). We also show here that (v) excitatory CA VII-dependent GABA signalling promotes eFS at P14, and the relevance of this finding is underscored by the observation (vi) that CA VII is expressed in the human cortex perinatally, well in advance of the age of 6 months when FS are first seen in children (Berg and Shinnar, 1996; Stafstrom, 2002).

Our previous work on rat hippocampal slices has demonstrated that the emergence of excitatory HCO_3_^−^-dependent GABA_A_R-mediated responses in CA1 neurons (Kaila et al, 1997) coincides with the onset of CA VII mRNA expression at around P10–12 (Ruusuvuori et al, 2004). However, these data demonstrate a correlation but not a cause–effect link between CA VII expression and GABAergic excitation because the possibility remained that some other, unidentified isoform might have been expressed in parallel with CA VII. The present data, which are based on the use of a novel CA VII KO mouse as well as a CA II KO (Lewis et al, 1988) and a CA II/CA VII double KO, clearly indicate that the two isoforms are sequentially expressed. CA VII is the only cytosolic CA isoform during P10–P18, while the two isoforms are co-expressed starting at around P20. Given the steeply increasing interest in the physiology, pathophysiology and pharmacology of individual CA isoforms (Supuran, 2008), this kind of information is of immense value in the design and evaluation of experiments on neuronal CAs, and makes the hippocampal developmental time window of ∼P12–16 (see Figure 2) eminently suited for examining the properties of CA VII in isolation. This isoform is interesting in that it is mainly expressed in the brain; moreover, in brain parenchyma its expression is restricted to neurons. The ubiquitous CA II isoform is expressed in both neurons and glia and abundantly outside the CNS (Ghandour et al, 1980; Wang et al, 2002; Supuran, 2008). Notably, the complete absence of CA activity in pyramidal neurons before P10 and in CA II/CA VII double KO neurons at all ages clearly shows that mature pyramidal neurons express only these two cytosolic isoforms.

### Somatodendritic developmental expression of CA II and CA VII in pyramidal neurons

As demonstrated by the present somatic pH_i_ recordings and excitatory dendritic GABA_A_R responses, the subcellular somatodendritic expression patterns of CA VII and CA II do not differ from each other. Moreover, both isoforms were equally effective in promoting somatic CO_2_-dependent pH shifts separately from, and in combination with, each other. The intrinsic catalytic rates of CA II and CA VII are extremely high (Earnhardt et al, 1998), and hence it is obvious that the present assay (see Figure 2) does not reflect the kinetic properties of these two isoforms. Nevertheless, our data strongly suggest that neither isoform acting in isolation would be rate-limiting for pH_i_ changes during major physiological and pathophysiological acid-base shifts.

A possible explanation for the presence of two cytosolic CA isoforms after P20 is that they show differences in their biochemical functions other than (de)hydration of CO_2_, such as esterase/phosphatase activity (Innocenti et al, 2008). Furthermore, the HCO_3_^−^ transporters make a significant contribution to neuronal pH regulation (Hentschke et al, 2006; Jacobs et al, 2008; Sinning et al, 2011). By forming isoform-specific metabolons with distinct acid-base transporters, CA II and VII may contribute to developmentally and spatially distinct pH_i_ microdomains (Sterling et al, 2001; Becker and Deitmer, 2007; see also Boron, 2010; Stridh et al, 2012). Moreover, recent work suggests that CA VII acts as scavenger of oxygen radicals (Truppo et al, 2012), which fits well with the large increase in cortical ocidateve energy metabolism taking place at the time of onset of CA VII expression (Erecinska et al, 2004).

### GABAergic excitation is promoted by both CA VII and CA II

Work on single crayfish muscle fibres and neurons originally demonstrated that the net efflux of intracellular HCO_3_^−^ across GABA_A_Rs leads to GABAergic depolarization by exerting a direct depolarizing action and, indirectly, by facilitating conductive uptake of Cl^−^ (Kaila and Voipio, 1987; Kaila et al, 1989; Voipio et al, 1991). The GABA_A_R-mediated CA-dependent acidosis is readily explained by the fact that HCO_3_^−^ is a substrate of cytosolic CAs, and a conductive net loss of HCO_3_^−^ will by necessity shift the balance of the CA-catalysed (de)hydration reaction to more acidic pH_i_ values (Kaila et al, 1990; Luckermann et al, 1997). Accordingly, in mammalian neurons and especially in their dendrites, which have a much larger surface-to-volume ratio (Qian and Sejnowski, 1990), CA activity is generally thought to be necessary for maintaining the supply of intracellular HCO_3_^−^ that drives GABAergic depolarizing and excitatory responses (Pasternack et al, 1993; Viitanen et al, 2010). In the present work, we addressed the roles of the two isoforms in the generation of HCO_3_^−^-dependent GABAergic depolarization. Our data show that CA VII has a unique role in promoting Cl^−^ accumulation (Viitanen et al, 2010) and consequent depolarizing GABAergic responses during P10–18, a time window that is known to be associated with a heightened proneness to epileptogenesis in rodents (Swann et al, 1993; Gloveli et al, 1995). The co-expression of isoforms VII and II starting at P20 had no obvious effect on the GABA_A_R-mediated depolarization.

### Role of CA VII-dependent excitatory GABA transmission in the generation of eFS

The molecular and neuronal network mechanisms underlying eFS have been extensively studied in various inbred and mutant mouse strains at the age of P14 (van Gassen et al, 2008; Wimmer et al, 2010). This is a very intriguing time point in the present context, because it enables a comparison of susceptibility to eFS of mice that have, or are completely devoid of, cytosolic CA in pyramidal neurons—that is, the WT and CA VII KO mice, respectively.

We have previously shown that a respiratory alkalosis promotes the generation of eFS in rodents (Schuchmann et al, 2006), and a recent retrospective clinical study has shown that a respiratory alkalosis is characteristic of FS in children (Schuchmann et al, 2011). Notably, the present data on breath rates and direct measurements of blood pH show that CA VII plays no role in hyperthermia-induced respiratory alkalosis in rodents. Here, one should recall that the non-catalysed (de)hydration of CO_2_ has a half-time of 8 s (Geers and Gros, 2000) at a typical physiological temperature. CA activity is therefore not likely to be important for the extracellular and consequent intracellular pH changes (*cf.* Ruusuvuori et al, 2010) that are associated with the slow hyperthermia-induced respiratory alkalosis, which has a time-to-seizure induction of about 20 min. In contrast, intraneuronal CA activity is needed for the fast replenishment of HCO_3_^−^ and consequent net uptake of Cl^−^, which are key mechanisms in the generation of excitatory HCO_3_^−^-dependent GABAergic responses (Ruusuvuori et al, 2004; Viitanen et al, 2010). Indeed, the present EEG data indicate that CA VII plays a central role in the generation of electrographic seizure activity associated with eFS in the cortex of P14 mice. The hypothesis that CA VII facilitates eFS by boosting GABAergic excitation is supported by two lines of evidence: (i) the behavioural eFS in P14 rats were enhanced by a low dose of diazepam, which is likely to act solely on GABA_A_Rs (Eghbali et al, 1997), while a high concentration blocked seizures; and (ii) parallel *in vitro* experiments showed that the excitatory GABAergic response was markedly potentiated by 1 μM diazepam. Interestingly, a recent paper has demonstrated an enhancement of GABAergic transmission in the GABRG2^R43/Q43^ knock-in mouse model of human FS (Hill et al, 2011).

The above data point to a facilitatory role for CA VII activity and consequent GABAergic excitation in the generation of FS. Importantly, the difference between genotypes in eFS susceptibility cannot be attributed to a difference in body temperature at the onset of the seizures. The dual effects of diazepam *in vivo* raise the intriguing possibility (see also Schuchmann et al, 2011) that the high therapeutic concentrations needed for suppression of FS in children exert their action via suppression of breathing rather than acting directly on the neuronal networks that are immediately responsible for the generation of FS. Indeed, it is a well-known fact that high concentrations of diazepines interfere with respiratory functions, especially in children (Orr et al, 1991; Tasker, 1998; Norris et al, 1999; Appleton et al, 2008). The expected outcome is block of the respiratory alkalosis with consequent suppression of FS.

Importantly, the expression of CA VII in the human cortex and hippocampus, as demonstrated in the exon array and droplet digital PCR analyses, already starts perinatally, while FS are typically seen in children at 6 months of age with the incidence peaking at 1.5 years (Berg and Shinnar, 1996; Stafstrom, 2002). CA inhibitors have been used for decades as anticonvulsant agents, but their side effects, including interference with bone formation (Lehenkari et al, 1998), preclude their long-term use in children. Notably, CA VII shows an expression profile that is mainly neuronal. Thus, designing isoform-specific inhibitors of CA VII has much potential as a novel approach in the treatment of FS and possibly of other epileptiform syndromes.

## Materials and methods

Animal experiments were approved by the responsible local institutions and complied with the regulations of the US National Institutes of Health and with those of the Society for Neuroscience (USA). Researchers were blind to the genotype during experiments and data analysis. Human tissues were collected as before (Kang et al, 2011) after obtaining parental or next-of-kin consent and with approval by the relevant institutional review boards.

### Generation of CA VII KO mice

The *Car7* gene was disrupted by homologous recombination in ES cells and blastocyst injection as detailed in Results and in Supplementary Materials and methods.

### Northern analysis

Northern blot analysis was performed with RNA isolated from various tissues of adult mouse as described previously (Jacobs et al, 2008). The probe corresponded to nucleotides 210–492 of the *Car7* reference sequence (NM_053070.3). *Gapdh* served as a loading control.

### Antibody generation and western analysis

The CA VII antiserum was raised in rabbits against the epitope DNFRPPQPLKGRVVK (amino acids 245–259) of the CA VII protein (accession number NM_053070.3) coupled via an N-terminal cysteine to keyhole limpet haemocyanin and affinity-purified. For western blots shown in Figure 1, 10 μg protein lysates from whole hippocampi or from cultured cells was separated on reducing 12% SDS–polyacrylamide gels and blotted on to a PVDF membrane as described in Sinning et al, 2011. Blots were probed with the rabbit CA VII antibody at a dilution of 1:250. Detection was performed with the chemiluminescence ECL Kit (Amersham Biosciences). Comparison of immunostainings for CA VII on brain sections from WT and KO mice revealed that our antibody was not suitable for immunolocalization studies because of unspecific staining. Mixed neuron/glia cultures or glial cell cultures were performed as described previously (Boettger et al, 2003). For additional western blots, see Supplementary Materials and methods.

### Generation of CA II/VII double KO mice

Animals heterozygous for both CA II and CA VII alleles were used to generate CA II/VII double KO mice. For more details on the CA II/VII double KO and genotyping, see Supplementary Materials and methods.

### Electrophysiology and pH_i_ fluorescence imaging

For details on electrophysiological recordings and BCECF fluorescence imaging of pH_i_ from hippocampal CA1 pyramidal neurons, see Supplementary Materials and methods.

### EEG recordings and eFS induction

In P14 rat pups, eFS were induced using a pre-heated chamber as described elsewhere (Schuchmann et al, 2006). For intracranial EEG recordings and for detection of behavioural hyperthermia effects in P13–14 mice, the temperature of the chamber was set to 43±1°C. For details see Supplementary Materials and methods.

### Blood analysis

GEM 4000 (Instrumentation Laboratory, Bedford, MA) was used in the analysis of trunk blood electrolytes and pH. Arteriovenous blood (80 μl) from control mice and from mice that underwent hyperthermia was collected after decapitation.

### CA VII detection in developing human brain

Exon array analysis was performed as previously described (Kang et al, 2011). For secondary validation using droplet digital PCR analysis, an aliquot of the total RNA that was previously extracted from each brain region was used. For more details, see Supplementary Materials and methods.

### Statistical analysis

All numerical data are expressed as mean±s.e.m. Statistical analyses of data were performed with two-tailed Student’s *t*-test or with ANOVA, Bonferroni as appropriate. *P*-values are given in the figure legends.

## Supplementary Material

Supplementary Information

Review Process File

## References

[b1] Aamand R, Skewes J, Moller A, Fago A, Roepstorff A (2011) Enhancing effects of acetazolamide on neuronal activity correlate with enhanced visual processing ability in humans. Neuropharmacology 61: 900–908 21736887 10.1016/j.neuropharm.2011.06.010

[b2] Alger BE, Nicoll RA (1982) Pharmacological evidence for two kinds of GABA receptor on rat hippocampal pyramidal cells studied *in vitro*. J Physiol 328: 125–141 7131310 10.1113/jphysiol.1982.sp014256 PMC1225650

[b3] Appleton R, Macleod S, Martland T (2008) Drug management for acute tonic-clonic convulsions including convulsive status epilepticus in children. Cochrane Database Syst Rev CD001905 18646081 10.1002/14651858.CD001905.pub2

[b4] Becker HM, Deitmer JW (2007) Carbonic anhydrase II increases the activity of the human electrogenic Na^+^/HCO_3_^−^ cotransporter. J Biol Chem 282: 13508–13521 17353189 10.1074/jbc.M700066200

[b5] Berg AT, Shinnar S (1996) Complex febrile seizures. Epilepsia 37: 126–133 8635422 10.1111/j.1528-1157.1996.tb00003.x

[b6] Boettger T, Rust MB, Maier H, Seidenbecher T, Schweizer M, Keating DJ, Faulhaber J, Ehmke H, Pfeffer C, Scheel O, Lemcke B, Horst J, Leuwer R, Pape HC, Volkl H, Hübner CA, Jentsch TJ (2003) Loss of K-Cl co-transporter KCC3 causes deafness, neurodegeneration and reduced seizure threshold. EMBO J 22: 5422–5434 14532115 10.1093/emboj/cdg519 PMC213773

[b7] Bootorabi F, Janis J, Smith E, Waheed A, Kukkurainen S, Hytonen V, Valjakka J, Supuran CT, Vullo D, Sly WS, Parkkila S (2010) Analysis of a shortened form of human carbonic anhydrase VII expressed *in vitro* compared to the full-length enzyme. Biochimie 92: 1072–1080 20493921 10.1016/j.biochi.2010.05.008

[b8] Boron WF (2010) Evaluating the role of carbonic anhydrases in the transport of HCO3--related species. Biochim Biophys Acta 1804: 410–421 19879980 10.1016/j.bbapap.2009.10.021 PMC4699190

[b9] Casey JR, Grinstein S, Orlowski J (2010) Sensors and regulators of intracellular pH. Nat Rev Mol Cell Biol 11: 50–61 19997129 10.1038/nrm2820

[b10] Chesler M (2003) Regulation and modulation of pH in the brain. Physiol Rev 83: 1183–1221 14506304 10.1152/physrev.00010.2003

[b11] Dube C, Chen K, Eghbal-Ahmadi M, Brunson K, Soltesz I, Baram TZ (2000) Prolonged febrile seizures in the immature rat model enhance hippocampal excitability long term. Ann Neurol 47: 336–344 10716253 PMC3139468

[b12] Dube C, Vezzani A, Behrens M, Bartfai T, Baram TZ (2005) Interleukin-1beta contributes to the generation of experimental febrile seizures. Ann Neurol 57: 152–155 15622539 10.1002/ana.20358 PMC2909879

[b13] Earnhardt JN, Qian MZ, Tu CK, Lakkis MM, Bergenhem NCH, Laipis PJ, Tashian RE, Silverman DN (1998) The catalytic properties of murine carbonic anhydrase VII. Biochemistry 37: 10837–10845 9692974 10.1021/bi980046t

[b14] Eghbali M, Curmi JP, Birnir B, Gage PW (1997) Hippocampal GABA(A) channel conductance increased by diazepam. Nature 388: 71–75 9214504 10.1038/40404

[b15] Enyedi P, Czirjak G (2010) Molecular background of leak K^+^ currents: Two-pore domain potassium channels. Physiol Rev 90: 559–605 20393194 10.1152/physrev.00029.2009

[b16] Erecinska M, Cherian S, Silver IA (2004) Energy metabolism in mammalian brain during development. Prog Neurobiol 73: 397–445 15313334 10.1016/j.pneurobio.2004.06.003

[b17] Farrant M, Kaila K (2007) The cellular, molecular and ionic basis of GABAA receptor signalling. Prog Brain Res 160: 59–87 17499109 10.1016/S0079-6123(06)60005-8

[b18] Fujiwara-Tsukamoto Y, Isomura Y, Imanishi M, Fukai T, Takada M (2007) Distinct types of ionic modulation of GABA actions in pyramidal cells and interneurons during electrical induction of hippocampal seizure-like network activity. Eur J Neurosci 25: 2713–2725 17459104 10.1111/j.1460-9568.2007.05543.x

[b19] Geers C, Gros G (2000) Carbon dioxide transport and carbonic anhydrase in blood and muscle. Physiol Rev 80: 681–715 10747205 10.1152/physrev.2000.80.2.681

[b20] Ghandour MS, Langley OK, Vincendon G, Gombos G, Filippi D, Limozin N, Dalmasso C, Laurent G (1980) Immunochemical and immunohistochemical study of carbonic anhydrase-II in adult rat cerebellum—a marker for oligodendrocytes. Neuroscience 5: 559–571 6769072 10.1016/0306-4522(80)90053-6

[b21] Gloveli T, Albrecht D, Heinemann U (1995) Properties of low Mg^2+^ induced epileptiform activity in rat hippocampal and entorhinal cortex slices during adolescence. Dev Brain Res 87: 145–152 7586496 10.1016/0165-3806(95)00069-p

[b22] Hentschke M, Wiemann M, Hentschke S, Kurth I, Hermans-Borgmeyer I, Seidenbecher T, Jentsch TJ, Gal A, Hübner CA (2006) Mice with a targeted disruption of the Cl^−^/HCO_3_^−^ exchanger AE3 display a reduced seizure threshold. Mol Cell Biol 26: 182–191 16354689 10.1128/MCB.26.1.182-191.2006 PMC1317631

[b23] Hill EL, Hosie S, Mulligan RS, Richards KL, Davies PJ, Dube CM, Baram TZ, Reid CA, Jones MV, Petrou S (2011) Temperature elevation increases GABA(A)-mediated cortical inhibition in a mouse model of genetic epilepsy. Epilepsia 52: 179–184 21219304 10.1111/j.1528-1167.2010.02914.x PMC3354703

[b24] Hille B (2001) Ionic channels of excitable membranes Third Edition. Sunderland, Massachusetts: Sinauer Associates,

[b25] Hirtz DG, Nelson KB (1983) The natural history of febrile seizures. Annu Rev Med 34: 453–471 6344771 10.1146/annurev.me.34.020183.002321

[b26] Innocenti A, Scozzafava A, Parkkila S, Puccetti L, De Simone G, Supuran CT (2008) Investigations of the esterase, phosphatase, and sulfatase activities of the cytosolic mammalian carbonic anhydrase isoforms I, II, and XIII with 4-nitrophenyl esters as substrates. Bioorg Med Chem Lett 18: 2267–2271 18353640 10.1016/j.bmcl.2008.03.012

[b27] Jacobs S, Ruusuvuori E, Sipila ST, Haapanen A, Damkier HH, Kurth I, Hentschke M, Schweizer M, Rudhard Y, Laatikainen LM, Tyynela J, Praetorius J, Voipio J, Hubner CA (2008) Mice with targeted Slc4a10 gene disruption have small brain ventricles and show reduced neuronal excitability. Proc Natl Acad Sci USA 105: 311–316 18165320 10.1073/pnas.0705487105 PMC2224208

[b28] Kaila K, Lamsa K, Smirnov S, Taira T, Voipio J (1997) Long-lasting GABA-mediated depolarization evoked by high- frequency stimulation in pyramidal neurons of rat hippocampal slice is attributable to a network-driven, bicarbonate-dependent K^+^ transient. J Neurosci 17: 7662–7672 9315888 10.1523/JNEUROSCI.17-20-07662.1997 PMC6793904

[b29] Kaila K, Pasternack M, Saarikoski J, Voipio J (1989) Influence of GABA-gated bicarbonate conductance on potential, current and intracellular chloride in crayfish muscle fibres. J Physiol 416: 161–181 2481729 10.1113/jphysiol.1989.sp017755 PMC1189209

[b30] Kaila K, Ransom BR (1998) pH and Brain Function New York: Wiley-Liss, Inc.,

[b31] Kaila K, Saarikoski J, Voipio J (1990) Mechanism of action of GABA on intracellular pH and on surface pH in crayfish muscle fibres. J Physiol 427: 241–260 1698980 10.1113/jphysiol.1990.sp018170 PMC1189929

[b32] Kaila K, Voipio J (1987) Postsynaptic fall in intracellular pH induced by GABA-activated bicarbonate conductance. Nature 330: 163–165 3670401 10.1038/330163a0

[b33] Kang HJ (2011) Spatio-temporal transcriptome of the human brain. Nature 478: 483–489 22031440 10.1038/nature10523 PMC3566780

[b34] Laorden ML, Carrillo E, Miralles FS, Puig MM (1990) Effects of diltiazem on hyperthermia induced seizures in the rat pup. Gen Pharmacol 21: 313–315 2341017 10.1016/0306-3623(90)90829-b

[b35] Lehenkari P, Hentunen TA, Laitala-Leinonen T, Tuukkanen J, Vaananen HK (1998) Carbonic anhydrase II plays a major role in osteoclast differentiation and bone resorption by effecting the steady state intracellular pH and Ca^2+^. Exp Cell Res 242: 128–137 9665810 10.1006/excr.1998.4071

[b36] Lewis SE, Erickson RP, Barnett LB, Venta PJ, Tashian RE (1988) N-ethyl-N-nitrosurea induced mutation at the mouse Car-2 locus - an animal model for human carbonic anhydrase II-deficiency syndrome. Proc Natl Acad Sci USA 85: 1962–1966 3126501 10.1073/pnas.85.6.1962 PMC279901

[b37] Luckermann M, Trapp S, Ballanyi K (1997) GABA- and glycine-mediated fall of intracellular pH in rat medullary neurons *in situ*. J Neurophysiol 77: 1844–1852 9114240 10.1152/jn.1997.77.4.1844

[b38] Magnotta VA, Heo HY, Dlouhy BJ, Dahdaleh NS, Follmer RL, Thedens DR, Welsh MJ, Wemmie JA (2012) Detecting activity-evoked pH changes in human brain. Proc Natl Acad Sci USA 109: 8270–8273 22566645 10.1073/pnas.1205902109 PMC3361452

[b39] Makani S, Chen HY, Esquenazi S, Shah GN, Waheed A, Sly WS, Chesler M (2012) NMDA receptor-dependent afterdepolarizations are curtailed by carbonic anhydrase 14: regulation of a short-term postsynaptic potentiation. J Neurosci 32: 16754–16762 23175829 10.1523/JNEUROSCI.1467-12.2012 PMC3514870

[b40] Meisler MH, Kearney J, Ottman R, Escayg A (2001) Identification of epilepsy genes in human and mouse. Annu Rev Genet 35: 567–588 11700294 10.1146/annurev.genet.35.102401.091142 PMC2765248

[b41] Munsch T, Pape HC (1999) Modulation of the hyperpolarization-activated cation current of rat thalamic relay neurones by intracellular pH. J Physiol 519 Pt 2: 493–504 10457065 10.1111/j.1469-7793.1999.0493m.x PMC2269522

[b42] Norris E, Marzouk O, Nunn A, McIntyre J, Choonara I (1999) Respiratory depression in children receiving diazepam for acute seizures: a prospective study. Dev Med Child Neurol 41: 340–343 10378761 10.1017/s0012162299000742

[b43] Orr RA, Dimand RJ, Venkataraman ST, Karr VA, Kennedy KJ (1991) Diazepam and intubation in emergency treatment of seizures in children. Ann Emerg Med 20: 1009–1013 1877765 10.1016/s0196-0644(05)82981-6

[b44] Pasternack M, Smirnov S, Kaila K (1996) Proton modulation of functionally distinct GABA_A_ receptors in acutely isolated pyramidal neurons of rat hippocampus. Neuropharmacology 35: 1279–1288 9014143 10.1016/s0028-3908(96)00075-5

[b45] Pasternack M, Voipio J, Kaila K (1993) Intracellular carbonic anhydrase activity and its role in GABA-induced acidosis in isolated rat hippocampal pyramidal neurones. Acta Physiol Scand 148: 229–231 8352034 10.1111/j.1748-1716.1993.tb09553.x

[b46] Pickkers P, Hughes AD, Russel FG, Thien T, Smits P (2001) *In vivo* evidence for K(Ca) channel opening properties of acetazolamide in the human vasculature. Br J Pharmacol 132: 443–450 11159693 10.1038/sj.bjp.0703825 PMC1572572

[b47] Qian N, Sejnowski TJ (1990) When is an inhibitory synapse effective? Proc Natl Acad Sci USA 87: 8145–8149 2236028 10.1073/pnas.87.20.8145 PMC54909

[b76] Ruusuvuori E, Kaila K (2013) Carbonic anhydrases and brain pH in the control of neuronal excitability. InCarbonic Anhydrase: Mechanism, Regulation, Links to Disease, and Industrial Applications Frost SC, McKenna R (eds), Springer (in press 10.1007/978-94-007-7359-2_14 24146384

[b48] Ruusuvuori E, Kirilkin I, Pandya N, Kaila K (2010) Spontaneous network events driven by depolarizing GABA action in neonatal hippocampal slices are not attributable to deficient mitochondrial energy metabolism. J Neurosci 30: 15638–15642 21084619 10.1523/JNEUROSCI.3355-10.2010 PMC6633692

[b49] Ruusuvuori E, Li H, Huttu K, Palva JM, Smirnov S, Rivera C, Kaila K, Voipio J (2004) Carbonic anhydrase isoform VII acts as a molecular switch in the development of synchronous gamma-frequency firing of hippocampal CA1 pyramidal cells. J Neurosci 24: 2699–2707 15028762 10.1523/JNEUROSCI.5176-03.2004 PMC6729533

[b50] Schuchmann S, Hauck S, Henning S, Gruters-Kieslich A, Vanhatalo S, Schmitz D, Kaila K (2011) Respiratory alkalosis in children with febrile seizures. Epilepsia 52: 1949–1955 21910730 10.1111/j.1528-1167.2011.03259.x

[b51] Schuchmann S, Schmitz D, Rivera C, Vanhatalo S, Salmen B, Mackie K, Sipilä ST, Voipio J, Kaila K (2006) Experimental febrile seizures are precipitated by a hyperthermia-induced respiratory alkalosis. Nat Med 12: 817–823 16819552 10.1038/nm1422 PMC1698875

[b52] Schuchmann S, Vanhatalo S, Kaila K (2009) Neurobiological and physiological mechanisms of fever-related epileptiform syndromes. Brain Dev 31: 378–382 19201562 10.1016/j.braindev.2008.11.011

[b53] Sinning A, Liebmann L, Kougioumtzes A, Westermann M, Bruehl C, Hubner CA (2011) Synaptic glutamate release is modulated by the Na^+^-driven Cl^−^/HCO_3_^−^ exchanger Slc4a8. J Neurosci 31: 7300–7311 21593314 10.1523/JNEUROSCI.0269-11.2011 PMC6622604

[b54] Spray DC, Harris AL, Bennet MVL (1981) Gap junctional conductance is a simple and sensitive function of intracellular pH. Science 211: 712–715 6779379 10.1126/science.6779379

[b55] Stafstrom CE (2002) The incidence and prevalence of febrile seizures. In:Febrile seizures Baram TZ, Shinnar S (eds). pp1–25.Academic Press: San Diego,

[b56] Staley KJ, Soldo BL, Proctor WR (1995) Ionic mechanisms of neuronal excitation by inhibitory GABA(A) receptors. Science 269: 977–981 7638623 10.1126/science.7638623

[b57] Sterling D, Reithmeier RAF, Casey JR (2001) A transport metabolon. Functional interaction of carbonic anhydrase II and chloride/bicarbonate exchangers. J Biol Chem 276: 47886–47894 11606574 10.1074/jbc.M105959200

[b58] Stridh MH, Alt MD, Wittmann S, Heidtmann H, Aggarwal M, Riederer B, Seidler U, Wennemuth G, McKenna R, Deitmer JW, Becker HM (2012) Lactate flux in astrocytes is enhanced by a non-catalytic action of carbonic anhydrase II. J Physiol 590: 2333–2351 22451434 10.1113/jphysiol.2011.220152 PMC3424756

[b59] Supuran CT (2008) Carbonic anhydrases: novel therapeutic applications for inhibitors and activators. Nat Rev Drug Discov 7: 168–181 18167490 10.1038/nrd2467

[b60] Swann JW, Smith KL, Brady RJ (1993) Localized excitatory synaptic-interactions mediate the sustained depolarization of electrographic seizures in developing hippocampus. J Neurosci 13: 4680–4689 7901349 10.1523/JNEUROSCI.13-11-04680.1993 PMC6576358

[b61] Tasker RC (1998) Emergency treatment of acute seizures and status epilepticus. Arch Dis Child 79: 78–83 9771259 10.1136/adc.79.1.78 PMC1717627

[b62] Thiry A, Dogne J, Supuran CT, Masereel B (2007) Carbonic anhydrase inhibitors as anticonvulsant agents. Curr Top Med Chem 7: 855–864 17504130 10.2174/156802607780636726

[b63] Tolner EA, Hochman DW, Hassinen P, Otahal J, Gaily E, Haglund MM, Kubova H, Schuchmann S, Vanhatalo S, Kaila K (2011) Five percent CO_2_ is a potent, fast-acting inhalation anticonvulsant. Epilepsia 52: 104–114 20887367 10.1111/j.1528-1167.2010.02731.x PMC3017646

[b64] Traynelis SF, Wollmuth LP, McBain CJ, Menniti FS, Vance KM, Ogden KK, Hansen KB, Yuan H, Myers SJ, Dingledine R (2010) Glutamate receptor ion channels: structure, regulation, and function. Pharmacol Rev 62: 405–496 20716669 10.1124/pr.109.002451 PMC2964903

[b65] Truppo E, Supuran CT, Sandomenico A, Vullo D, Innocenti A, Di FA, Alterio V, De SG, Monti SM (2012) Carbonic anhydrase VII is S-glutathionylated without loss of catalytic activity and affinity for sulfonamide inhibitors. Bioorg Med Chem Lett 22: 1560–1564 22277279 10.1016/j.bmcl.2011.12.134

[b66] van Gassen KL, Hessel EV, Ramakers GM, Notenboom RG, Wolterink-Donselaar IG, Brakkee JH, Godschalk TC, Qiao X, Spruijt BM, van NO, De Graan PN (2008) Characterization of febrile seizures and febrile seizure susceptibility in mouse inbred strains. Genes Brain Behav 7: 578–586 18363854 10.1111/j.1601-183X.2008.00393.x

[b67] Velisek L, Dreier JP, Stanton PK, Heinemann U, Moshe SL (1994) Lowering of extracellular pH suppresses low-Mg^2+^ induced seizures in combined entorhinal cortex-hippocampal slices. Exp Brain Res 101: 44–52 7843301 10.1007/BF00243215

[b68] Viitanen T, Ruusuvuori E, Kaila K, Voipio J (2010) The KCl -cotransporter KCC2 promotes GABAergic excitation in the mature rat hippocampus. J Physiol 588: 1527–1540 20211979 10.1113/jphysiol.2009.181826 PMC2876807

[b69] Voipio J, Kaila K (1993) Interstitial PCO^2^ and pH in rat hippocampal slices measured by means of a novel fast CO_2_/H^+^-sensitive microelectrode based on a PVC-gelled membrane. Pflugers Arch 423: 193–201 8391679 10.1007/BF00374394

[b70] Voipio J, Pasternack M, Rydqvist B, Kaila K (1991) Effect of gamma-aminobutyric acid on intracellular pH in the crayfish stretch-receptor neurone. J Exp Biol 156: 349–360 1711091 10.1242/jeb.156.1.349

[b71] Waldmann R, Champigny G, Bassilana F, Heurteaux C, Lazdunski M (1997) A proton-gated cation channel involved in acid-sensing. Nature 386: 173–177 9062189 10.1038/386173a0

[b72] Wang WG, Bradley SR, Richerson GB (2002) Quantification of the response of rat medullary raphe neurones to independent changes in pH_O_ and P_CO_2__. J Physiol 540: 951–970 11986382 10.1113/jphysiol.2001.013443 PMC2290275

[b73] Wilkins ME, Hosie AM, Smart TG (2005) Proton modulation of recombinant GABA(A) receptors: influence of GABA concentration and the beta subunit TM2-TM3 domain. J Physiol 567: 365–377 15946973 10.1113/jphysiol.2005.088823 PMC1474194

[b74] Williams RH, Jensen LT, Verkhratsky A, Fugger L, Burdakov D (2007) Control of hypothalamic orexin neurons by acid and CO2. Proc Natl Acad Sci USA 104: 10685–10690 17563364 10.1073/pnas.0702676104 PMC1965573

[b75] Wimmer VC, Reid CA, Mitchell S, Richards KL, Scaf BB, Leaw BT, Hill EL, Royeck M, Horstmann MT, Cromer BA, Davies PJ, Xu R, Lerche H, Berkovic SF, Beck H, Petrou S (2010) Axon initial segment dysfunction in a mouse model of genetic epilepsy with febrile seizures plus. J Clin Invest 120: 2661–2671 20628201 10.1172/JCI42219 PMC2912193

