# Supplementary material for: Neuronal carbonic anhydrase VII provides GABAergic excitatory drive to exacerbate febrile seizures
Source: EMBO J. 2013 Jul 23;32(16):2275–86. doi: 10.1038/emboj.2013.160 (PMC3746197; doi:10.1038/emboj.2013.160)
Supplement: Supplementary Information [file emboj2013160s1.pdf]

Supplemental information

Materials and methods

Generation of the CA VII KO mice. A clone isolated from a 129/SvJ mouse genomic λ library (Stratagene) was used to construct the targeting vector. A 12 kb fragment including exons 2 to 7 of the *Car7* gene was cloned into the pKO-V901 plasmid (Lexicon Genetics) with a phosphoglycerate kinase (pgk) promoter-driven diphtheria toxin A cassette. A pgk promoter-driven neomycin resistance cassette flanked by loxP-sites was inserted into the *Xho*I-site of intron 4. A third loxP-site and an additional *Eco*RI-site were inserted into the *Sac*II-site at the 3' end of the *Car7* gene. The construct was electroporated into R1 mouse embryonic stem cells. Neomycin resistant clones were analyzed by Southern blot using *Eco*RI and an external ~ 500 bp probe. Correctly targeted ES-cells were transfected with a plasmid expressing Cre-recombinase to remove the neomycin-cassette and exons 5, 6, and 7. Correctly recombined clones were identified with an internal probe by Southern blot analysis. Two independent embryonic stem cell clones were injected into C57BL/6 blastocysts to generate chimeras that were backcrossed with C57BL/6. Genotypes were either determined by Southern blot or by PCR on tail biopsy DNA. For PCR genotyping, the sense primer F1 (TGGAACGCCAAGAAGTACAGC) and the antisense primers R1 (ATGCCCTCACTGGGGAGATGG) and R2 (AGTCTTTATGGCAGAGACAGC) were used. The primer pair F1/R1 amplified a ~400 bp fragment of the wild-type allele, and the primer pair F1/R2 amplified a ~300 bp fragment of the knock-out allele. Mice were maintained on a 12 hour artificial light/dark cycle and provided with food and water *ad libitum*.

Matings between homozygous CA VII KO mice produced normal sized litters with viable pups. Hence, homozygous KO mating was used in addition to the heterozygous mating.

Generation of CA II/VII double KO mice. The heterozygous CA II and CA VII mice were used to generate mice devoid of both CA II and CA VII. The CA II/VII double KO animals were viable and born in the expected Mendelian ratio from heterozygous (~6.25 %, 9 out of the 194 genotyped animals) and homozygous CA VII/heterozygous CA II pairs (~12.5 %, 8 out of the 73 genotyped animals). The body weight of the CA II/VII KO's was smaller than that of their WT or heterozygous siblings as described previously for the CA II KO mice (Lewis et al.,

1988). For PCR genotyping of the CA II allele, primers WT-f (GTGAGTTACAGAGACAGAAG), WT-r (GGACTTTCTGAAGGACTTG), KO-f (GATTGGACCTGCCTCAT), and KO-r (GTTCTCTGGTTCTTGCATATG) were used to amplify fragments 237 bp (WT) and 163 bp (KO) in length.

Western analysis of muscle and liver tissue. For Western blotting, 10 µg protein lysates from hippocampus, skeletal muscle and liver were separated on reducing 15 % SDS-polyacrylamide gels and blotted onto a nitrocellulose membrane. Blots were probed with our rabbit CA VII antibody at a dilution of 1:250. Detection was done with Lasuser 3000 (Fujifilm) using chemoluminescence ECL-Kit (Pierce).

Solutions and slice preparation for pH_i and electrophysiological recordings. Transgenic mice (P5-46) or Wistar rats (P14) were decapitated, and the brains were dissected in ice cold (0-4 °C) solution. Coronal brain slices (350 µm) were cut with a vibrating blade microtome (Leica VT1000S). Dissection and preparation of the brain slices was done in modified standard solution containing (in mM) 87 NaCl, 70 sucrose, 2.5 KCl, 0.5 $CaCl_2$, 25 $NaHCO_3$, 1.1 NaH_2PO_4, 7 $MgSO_4$, and 25 D-glucose, equilibrated with 95% O_2, 5% CO_2. Slices were let to recover at 34°C for 30 minutes and then stored at room temperature. All recordings were performed in a submerged recording chamber at 32 ± 1°C, with one-sided perfusion, solution flow 4 ml/min. The standard solution contained (in mM) 124 NaCl, 3 KCl, 2 $CaCl_2$, 25 $NaHCO_3$, 1.1 NaH_2PO_4, 2 $MgSO_4$ and 10 D-glucose, equilibrated with 95% O_2, 5% CO_2, pH 7.4 at 32°C. In bicarbonate free, HEPES-buffered solution 25 mM $NaHCO_3$ was replaced with 10 mM NaOH, 10 mM NaCl and 20 mM HEPES (pH 7.4 at 32°C), and the solution was oxygenated with 100% O_2.

Detection of intrapyramidal carbonic anhydrase activity. In order to detect cytosolic CA activity, we exposed slices to nominally CO_2/HCO_3^- free HEPES solution for 100-200 s in the absence and presence of the membrane-permeant CA inhibitor acetazolamide (AZ 100 µM, Sigma-Aldrich, St.Louis, MO) and quantified the AZ induced suppression of the maximum rate of intracellular alkalinization (dpH_i/dt_{max}). Since AZ is known to inhibit both extra- and intracellular CA activity (Chesler, 2003), the control responses were done in the presence of the poorly permeant CA inhibitor benzolamide (BA, 10 µM) applied for 5 minutes before

withdrawal of CO_2/HCO_3^- to exclude effects mediated by inhibition of extracellular CAs. BA was a kind gift from Dr. E.R. Swenson (University of Washington, Seattle, WA).

pH_i was measured using the H^+-sensitive indicator BCECF. For the dye loading, brain slices were transferred to the recording chamber and perfused with standard solution. BCECF-AM (Invitrogen) was pressure-injected into the extracellular space of CA1 pyramidal layer using a conventional patch pipette, filled with 10 µM BCECF-AM in standard solution. In each slice, somatic pH_i was measured from 3-5 CA1 pyramidal neurons (see *Inset* in Figure 2*A*). Both loading and pH_i measurements were done in the continuous presence of TTX (1 µM).

Live-cell imaging was performed with an upright Zeiss Axio Scope II (60x water immersion objective n.a. 0.9). BCECF was excited with an OptoLED double wavelength light source (436 ± 10 nm and 500 ± 10 nm, Cairn, UK). Emitted light passed through a 535 ± 12.5 nm band pass filter and was registered with a PCO SensiCam CCD camera. The diameter of the region of interest (ROI) was about 3x5 µm, and the ROI was positioned at the site of the highest local signal. Ratiometric images were captured (at 0.1-0.2 Hz) and analyzed with WinFluor software (courtesy of Dr. John Dempster, University of Strathclyde, Glasgow, UK). For analysis the original pH-trace was smoothed with the Savitzky-Golay algorithm (20 data points frame, 2nd order polynomial) (Savitzky and Golay, 1964) and then differentiated with Origin software (OriginLab Corporation, Northampton, MA) using the equation:

$$\left(\frac{dpH_i}{dt}\right)_j = \frac{(pH_i)_{j+1} - (pH_i)_{j-1}}{t_{j+1} - t_{j-1}}$$

where *j* indicates the number of sample.

Electrophysiological recordings. Whole-cell patch-clamp recordings were performed from visually identified CA1 pyramidal cells. The glass micropipettes had a resistance of 4.5-6.0 MΩ when filled with intracellular solution consisting of (in mM) 124 K-gluconate, 5 KCl, 2 NaOH, 10 HEPES, 0.5 $CaCl_2$, 5 EGTA, 2 MgATP (pH adjusted to 7.3 with KOH). Recordings were made using an EPC 10 amplifier (HEKA Elektronik, Lambrecht/Pfalz, Germany) in the voltage-clamp or current-clamp mode and corrected for a calculated 15.0 mV liquid junction potential for whole-cell recordings (Barry, 1994). For field recordings, 4.5-6.0 MΩ glass microelectrodes were filled with the standard solution and placed in the stratum

pyramidale. The signal was high-pass filtered at 10 Hz. $GABA_A$ receptor-mediated synaptic responses were evoked in CA1 pyramidal neurons using a monopolar glass microelectrode filled with standard solution (resistance 4.5-6.0 MΩ, see Fig. 3) or, for a more intense $GABA_AR$ activation, with a bipolar metal electrode, placed in the border of *stratum radiatum* (*sr) and* stratum lacunosum-moleculare *(slm)* (Alger and Nicoll, 1982;Staley et al., 1995;Kaila et al., 1997). The intensity of high-frequency stimulation (HFS; 40 pulses at 100 Hz, given every 5 minutes) applied via the bipolar electrode (Kaila et al., 1997) was sufficient to evoke near-maximal (≥ 80 %) $GABA_AR$ responses, whereas the intensity of the monopolar stimulation (either single pulse stimulation at 15 s interval, or HFS) was adjusted to be slightly above the threshold of triggering $GABA_AR$-mediated responses with a negligible failure rate. $GABA_AR$-mediated responses were pharmacologically isolated by blocking ionotropic glutamate receptors with AP5 (40 µM) and CNQX (20 µM), and $GABA_B$ receptors with CGP-55845 (1 µM). $GABA_A$ receptors were blocked with PiTX (90 µM) or SR 95531 (50 µM), and DZP (1 µM) was used to enhance GABAergic transmission. Pressure microinjections of GABA (5 mM in standard solution) were applied via a glass capillary microelectrode (tip diameter 2–4 µm). Using Dodt gradient contrast, the apical dendrites of the CA1 pyramidal neurons were followed from site of recording to the border of *sr/slm*. The pressure injection pipette was placed to the border of *sr/slm* in the superficial layer of the slice. Brief pulses of pressure (18 pounds per square inch (psi), duration 8-100 ms) were given every 5 minutes. In these experiments TTX (1 µM) was used to prevent spiking, and CGP-55845 (1 µM) to block $GABA_B$ receptors. The drugs were from Tocris (Bristol, UK) except for diazepam which was a kind gift from Dr. Ari-Pekka Koivisto (Orion Pharma, Finland).

EEG measurements and FS induction.

Surgery and EEG recordings. On the day of experiments, typically two P13-14 animals from the same litter were studied. At this age CA VII KO mice were of comparable weight as their WT littermates (6.9 ± 0.2 g *n* = 11 vs. 6.4 ± 0.2 g, *n* = 9, respectively, *P* = 0.14, Student's *t*-test). For cortical EEG recordings ball-tipped Teflon-insulated silver wire electrodes (75 µm wire diameter, ball tip diameter ca. 1 mm; Advent Research Materials Ltd, Oxford, UK) were connected to microconnectors (Microtech Inc., Boothwyn, PA, USA) and fixed with dental acrylic. Electrodes were implanted under isoflurane anesthesia (maintained at 2-2.5 %).

Small craniotomies were made using a drill equipped with a 0.6 mm diameter carbide burr and electrodes were placed epidurally above right parietal cortex (AP 2.5 mm; ML 1.3 mm). A reference electrode was placed above cerebellum. The incision was sutured using 6-0 nylon monofilament. After surgery, mice recovered for two hours in a warm environment (35 °C) before the start of the experiment.

Before and during the induction of eFS, continuous cortical EEG and video recordings (Philips SPC 900 NC web camera; 25 frames/sec) were made from the freely moving mice. EEG signals were high-pass filtered (cut-off at 0.07 Hz) before 1000x amplification by a custom-made amplifier, and were sampled at 1 kHz using a 16-bit data acquisition interface and Labview software (National Instruments, Austin, Tx; custom-built functions courtesy of T. Maila).

Seizure induction. Seizures were induced using a heated chamber as described previously for neonatal rats (Schuchmann et al., 2006;2008). In parallel with the EEG and video recordings we monitored rectal temperature with a mouse rectal probe (BAT-10; Physitemp, Science Products GmbH, Hofheim, Germany) and breath rate with a piezo crystal sensor placed on the abdomen (Pico Movement Sensor; Temec, Kerkrade, Netherlands) (Schuchmann et al., 2008). After baseline recording at 30-34 ^{o}C for 10 minutes, pups were moved to a pre-heated chamber kept at 43 ± 1 ^{o}C for mice and at 48 ± 1 ^{o}C when rat pups were used. Animals were exposed to hyperthermia until their body temperature reached a critical high value of 42.5-43 ^{o}C (Dubé and Baram, 2006), after which the experiment was terminated and animals decapitated.

To test the effect of CO_2 on seizure generation, the heated chamber was supplied with a constant flow of pre-heated (43-44 °C) gas mixture of 5% CO_2, 19% O_2, and 76% N_2.

In experiments where diazepam was used (Stesolid Novum [Actavis], 5 mg/ml, diluted in 0.9% NaCl, injection volume 100 µl) the drug was given intraperitoneally (i.p.) at 50 µg/kg, 150 µg /kg or 2.5 mg/kg dose 15 minutes before the onset of hyperthermia. An equal volume of saline was injected into control animals.

Data analysis. EEG, breathing, temperature and video-recordings were analyzed off-line using Diadem 10.0 software (National Instruments, Austin, Tx). Electrographical seizure activity was recognized as bursts of regular spikes with amplitude values around 150-600 µV which is at least 3x standard deviation of baseline EEG amplitude with durations of 10

seconds up to 1 minute. Breath rate (breaths/min) was analyzed in the baseline period and hyperthermia. During hyperthermia, for WT animals the breath rate was analyzed within the minute just before EEG seizure onset, on average around the 24th minute (see Results). For CA VII KO mice, the breathing rate during hyperthermia was analyzed around the 24th minute after the start of hyperthermia. Detection of movement artefacts in the EEG traces was done using the piezo-transducer signal (see above) and the video.

Blood samples. To measure hyperthermia induced change in blood pH and electrolytes, we collected blood samples from control WT and CA VII KO mice and from WT and CA VII KO mice that were exposed to hyperthermia. From each animal 80 µl of blood was collected after decapitation into plastic capillaries (1.9x54 mm) with lyophilised lithium-heparin 100 I.U./ml (Sanguis Counting GmbH, Numbrecht, Germany) and pH was measured with GEM Premier 4000 device (Instrumentation Laboratory, USA).

From control animals the sample was collected after breathing rate and rectal temperature were recorded for 10 minutes. Animals that underwent hyperthermia were placed in a pre-heated chamber (43 ± 1 ^{o}C) after baseline measurement of breathing rate and body temperature. The blood sample was collected after the body temperature reached 41.5 ^{o}C. In experiments with EEG recordings all WT mice had developed seizures at this point.

Detection of CA VII in the developing human neorcortex and hippocampus.

Exon array analysis was performed as previously described (Kang et al., 2011). Briefly, total RNA was isolated from specific brain regions of neocortex and hippocampus using a non-phenolic procedure (RNeasy Plus Mini Kit, Qiagen), followed by DNase treatment (TURBO DNase, Ambion). Optical density values at 260/280 were consistently above 1.9 (NanoDrop, Thermo Scientific), and the values of RNA integrity were selected above 5 (RIN>5, Agilent Bioanalyzer). Synthesized cDNA (5.5 µg) using WT Expression kit (Ambion) was labeled and loaded onto individual Affymetrix Human Exon 1.0 ST arrays. Microarrays were hybridized at 45 °C for 16–24 hours, washed and stained using an Affymetrix FS450 fluidics station, according to manufacturer recommendations. Microarrays were scanned on a GeneChip Scanner 3000 and visually inspected for hybridization artifacts. The raw image files (.DAT files) were analysed using Affymetrix GeneChip Operating Software to generate .CEL files.

Droplet digital PCR. An aliquot of the total RNA that was previously extracted from each brain region was used for secondary validation using droplet digital PCR analysis.

One ug of total RNA was used for cDNA synthesis using oligo dT primers and SuperScript III First-strand synthesis Supermix (Invitrogen), and subsequently diluted with nuclease-free water to 1 ng/ul cDNA. Gene-specific high-melt temperature primers and FAM-probes for genes of interest were designed using PrimerQuest^{SM} (http://www.idtdna.com/Scitools/Applications/Primerquest) and expressed sequence information obtained from GenBank (NCBI). PCR reactions were conducted on the QX100 Droplet Digital^{TM} PCR system (Bio-Rad) according to manufacturer recommendations. Briefly, the reaction mixture containing sample cDNA, primers and probe was partitioned into about 20,000 droplets in oil through the QX100 Droplet Generator. After PCR amplification (95'C 10 min; 40 cycles of (94'C 30 sec, 57'C 60 sec); 98'C 2 min), each droplet provided a positive or negative fluorescent signal indicating the target gene was present or not present after partitioning. Positive and negative droplets were counted in the QX100 Droplet Reader and the software calculated the concentration of target gene as copies per microliter. The copy number of *CA VII* was normalized to the housekeeping gene *Gapdh*.

References

Alger BE, Nicoll RA (1982) Pharmacological evidence for two kinds of GABA receptor on rat hippocampal pyramidal cells studied in vitro. J Physiol 328:125-141.

Barry PH (1994) JPCalc, a software package for calculating liquid junction potential corrections in patch-clamp, intracellular, epithelial and bilayer measurements and for correcting junction potential measurements. J Neurosci Methods 51:107-116.

Chesler M (2003) Regulation and modulation of pH in the brain. Physiol Rev 83:1183-1221.

Dubé CM, Baram TZ (2006) Complex febrile seizures - An experimental model in immature rodents. In: Models of seizures and epilepsy (Pitkanen A, Schwartzkroin PA, Moshe SL, eds), pp 333-340. Elsevier Academic Press.

Kaila K, Lamsa K, Smirnov S, Taira T, Voipio J (1997) Long-lasting GABA-mediated depolarization evoked by high- frequency stimulation in pyramidal neurons of rat hippocampal slice is attributable to a network-driven, bicarbonate-dependent K+ transient. J Neurosci 17:7662-7672.

Kang HJ, et al. (2011) Spatio-temporal transcriptome of the human brain. Nature 478:483-489.

Lewis SE, Erickson RP, Barnett LB, Venta PJ, Tashian RE (1988) N-ethyl-N-nitrosurea induced mutation at the mouse Car-2 locus - an animal model for human carbonic anhudrase II-deficiency syndrome. Proc Natl Acad Sci U S A 85:1962-1966.

Savitzky A, Golay MJE (1964) Smoothing and differentation of data by simplified least squares procedures. Anal Chem 36:1627-1639.

Schuchmann S, Schmitz D, Rivera C, Vanhatalo S, Salmen B, Mackie K, Sipila ST, Voipio J, Kaila K (2006) Experimental febrile seizures are precipitated by a hyperthermia-induced respiratory alkalosis. Nat Med 12:817-823.

Schuchmann S, Tolner EA, Marshall P, Vanhatalo S, Kaila K (2008) Pronounced increase in breathing rate in the "hair dryer model" of experimental febrile seizures. Epilepsia 49:926-928.

Staley KJ, Soldo BL, Proctor WR (1995) Ionic mechanisms of neuronal excitation by inhibitory GABA(A) receptors. Science 269:977-981.

Figure S1 Ruusuvuori et *al.*

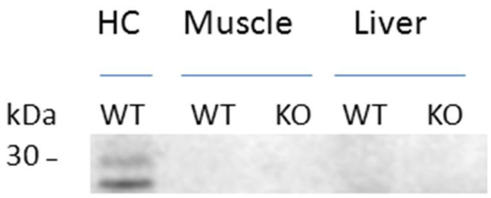

Supplemental Figure S1

CA VII protein is not present in skeletal muscle or liver. In agreement with the Northern analysis shown in Fig. 1B, Western analysis of adult WT mouse muscle and liver does not show any CA VII protein expression while a robust signal is seen in WT hippocampal tissue (HC) (see also Fig. 1C).

Figure S2 Ruusuvuori et *al.*

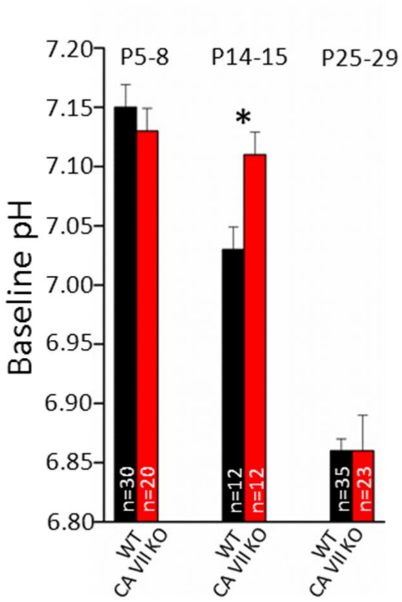

Supplemental Figure S2

Expression of cytosolic carbonic anhydrase changes somatic pH in CA1 pyramidal neurons. Intraneuronal pH measurements were performed with BCECF in slice preparations from P5-29 WT and CA VII KO mice. At P5-8 the baseline pH_i was similar in neurons from WT and CA VII KOs (mean + SEM; P = 0.5). In WT mice expression of CA VII starts at around P10 and causes an intraneuronal acidification (P14-15). In CA VII KO neurons pH_i remained at significantly more alkaline level (P = 0.011). This pH-difference is not seen in more mature neurons (P25-29) after the onset of CA II expression (P = 0.78). Numbers in bar diagrams denote the number of cells. *P*-values are based on Student's *t*-test (*P < 0.05).

Figure S3 Ruusuvuori et *al.*

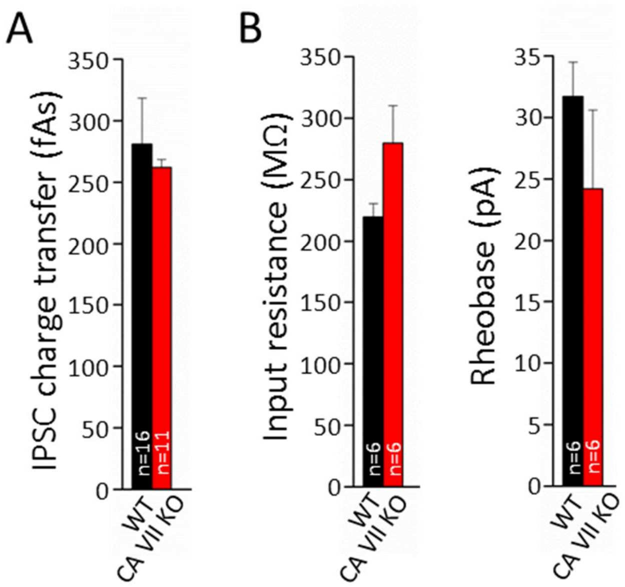

Supplemental Figure S3

The single inhibitory postsynaptic currents and the intrinsic excitability of CA1 pyramidal neurons are similar in WT and CA VII KO at P12-16. (A) The apparent charge transfer associated with single inhibitory postsynaptic currents (IPSCs) did not differ between the two genotypes (mean + SEM; P = 0.75). Measurements were made under voltage clamp conditions (V_m -70 mV) with minimal stimulation delivered to distal dendrites at 0.25 Hz. (B) The input resistance (R_{in}; P = 0.09) and the rheobase (P = 0.31) were comparable in the WT and CA VII KOs neurons. Numbers in bar diagrams denote the number of cells and P-values are based on Student's *t*-test.

Figure S4 Ruusuvuori et *al.*

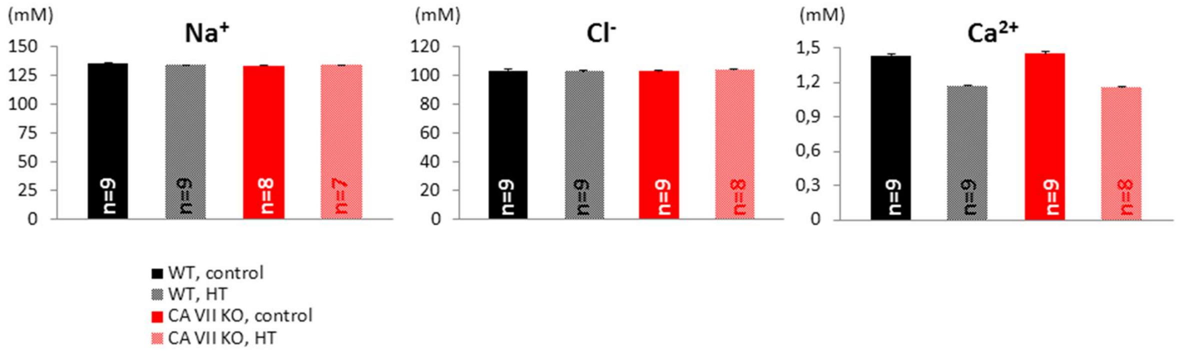

Supplemental Figure S4

Blood electrolyte values do not show genotype-specific differences in control or under hyperthermia. Under control conditions blood electrolyte levels in WT and CA VII KO mice had almost identical mean values and overlapping SEM values (Na^{+}: 135±0.8 mM vs. 134±0.4 mM; Cl^{-}: 103±0.8 mM vs. 103±0.4 mM; Ca^{2+}: 1.43±0.02 mM vs. 1.45±0.02 mM, respectively). Calcium, but not sodium and chloride, underwent a quantitatively identical decrease in hyperthermia (HT) in WT and CA VII KO mice (Ca^{2+} under hyperthermia 1.17±0.01 mM and 1.15±0.01, respectively). Values are given as mean + SEM, the number of animals is indicated in the bar diagram.

Figure S5 Ruusuvuori et *al.*

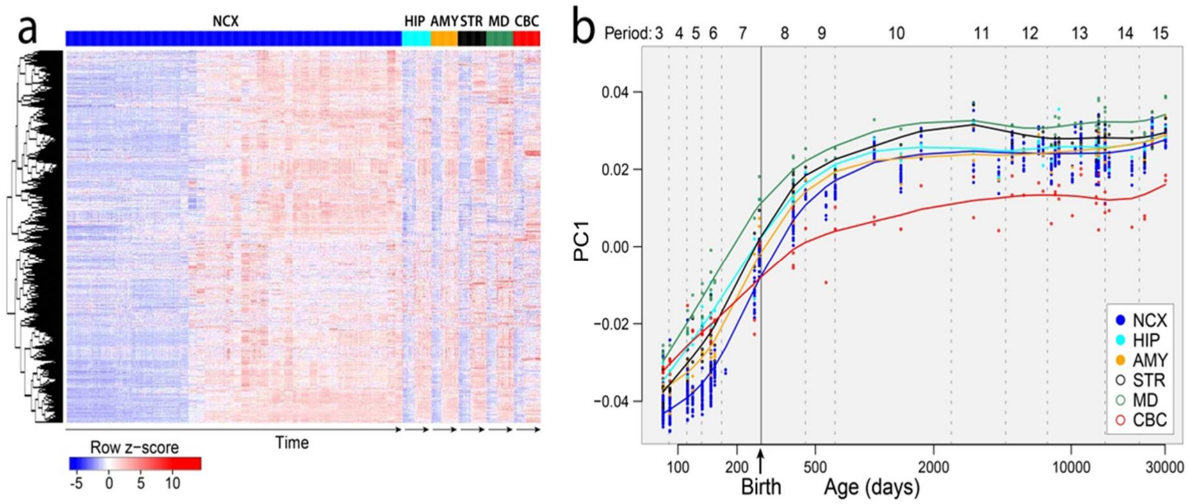

Supplemental Figure S5

WGCNA Module 2. M2 was associated with a progressive increase in gene expression across all regions starting at the embryonic period. (A) Heat map of genes in M2 after hierarchical clustering showing the temporally co-expressed pattern is consistent across all regions. The expression values for each gene were ordered first by brain regions, then by age, and last by neocortex areas. (B) The spatio-temporal pattern of M2 was summarized using PCA analysis. PC1 was plotted against age, after being grouped and color-coded according to brain regions. Neocortex (NCX), hippocampus (HIP), amygdala (AMY), striatum (STR), mediodorsal nucleus of the thalamus (MD), and cerebellar cortex (CBC). The pattern was summarized by the smoothed curves of PC1 values. Dashed lines represent division between periods of the development, and the solid line separates prenatal from postnatal periods
